# Mediation of coping style between academic self-efficacy and academic stress in middle school students

**DOI:** 10.3389/fpsyg.2025.1496528

**Published:** 2025-09-22

**Authors:** Pu Sun, Lifang Wang, Ling Yan

**Affiliations:** ^1^Capital University of Physical Education and Sports, Emerging Interdisciplinary Platform for Medicine and Engineering in Sports (EIPMES), Beijing, China; ^2^Department of Hexi University, Zhangye, China

**Keywords:** middle school students, academic stress, self-efficacy, coping strategies, mediation

## Abstract

**Objective:**

To explore the mediating role of coping styles between academic self-efficacy and academic stress among middle school students and to provide insights into potential intervention strategies to alleviate academic stress.

**Methods:**

A total of 2,720 middle school students participated in the survey, which utilized the Academic Self-Efficacy Scale, Academic Pressure Scale, and Simplified Coping Style Questionnaire. The sample included 1,336 boys (49.1%) and 1,384 girls (50.9%), with ages ranging from 11 to 18 years and an average age of 14.48 ± 1.47 years.

**Results:**

Academic stress was negatively correlated with academic self-efficacy and positive coping style (*r* = −0.37, −0.3, *p* < 0.001), and positively correlated with negative coping style (*r* = 0.32, *p* < 0.001). Both coping styles significantly mediated the relationship between academic self-efficacy and academic stress, with positive and negative coping accounting for 47.38 and 18% of the total effect, respectively.

**Conclusion:**

Academic self-efficacy has both direct and indirect effects on academic stress, with coping styles playing a critical mediating role. These findings suggest that fostering academic self-efficacy and encouraging positive coping strategies can effectively alleviate academic stress, providing insights for intervention programs aimed at promoting student wellbeing.

## Introduction

Academic stress refers to a subjective response experienced by learners arising from academic-related environments and demands that exceed individual coping abilities or pose a threat (Folkman, 2013). Under the context of increasing educational competition, academic stress has become a primary source of stress for middle school students (Liu, 2016). Factors contributing to academic stress include high expectations for academic performance, intense competition for higher education opportunities, and high expectations from families (Ma and Ni, 2014). As a pervasive and enduring source of stress, academic stress directly impacts students’ performance in various areas of their lives (Gao, 2023).

Moderate academic stress can stimulate students’ learning motivation and initiative. However, excessive academic stress may lead to adverse reactions such as aversion to learning, excessive fatigue, depression, and anxiety, ultimately affecting academic performance, mental health, and social adaptability (Yu and Yan, 2005). This not only negatively impacts academic performance and overall health but also impedes students’ social adaptability and long-term development (Kennett et al., 2021).

Academic self-efficacy refers to a learner’s evaluation of their confidence in successfully completing academic tasks using their existing abilities and skills. It reflects a learner’s belief in their academic capabilities and serves as an extension of self-efficacy within academic settings (Bandura and Wessels, 1997). Academic self-efficacy encompasses two dimensions: self-efficacy in learning abilities and self-efficacy in learning behaviors (Schunk, 1991). This construct illustrates learners’ attitudes toward challenging academic tasks, their level of effort in learning, and their use of effective learning strategies and metacognitive approaches. It is a crucial indicator of their confidence and sense of competence in academic domains (Honicke and Broadbent, 2016). Students with high academic self-efficacy are more capable of handling learning tasks, willing to face academic challenges (Honicke et al., 2023), and tend to exhibit better academic performance (Zimmerman, 2000). In contrast, low academic self-efficacy may undermine learning motivation and trigger anxiety or other emotional issues (Pajares and Schunk, 2004). Therefore, academic self-efficacy not only significantly influences students’ academic performance but also plays a critical role in their ability to cope with academic stress.

Although numerous studies have examined the relationship between academic self-efficacy and academic stress, the psychological mechanisms underlying the predictive effect of academic self-efficacy on academic stress—particularly the mediating role of coping style—remain underexplored. Moreover, as middle school students are at a critical stage of both academic and psychological development, large-scale empirical investigations into the dynamic relationships among academic self-efficacy, coping style, and academic stress in this population are still insufficient. By introducing coping style as a mediating variable, the present study not only enriches the literature on the relationship between academic self-efficacy and academic stress but also provides empirical support for psychological interventions and stress management practices in educational contexts.

## Theoretical framework and hypotheses

When understanding the relationship between academic self-efficacy and academic stress, the *Cognitive Appraisal Theory of Stress* provides a robust theoretical framework. Proposed by Lazarus and Folkman in 1984, this theory suggests that stress is a cognitive process in which individuals evaluate stressors to determine whether they pose a threat or a challenge. This evaluation process involves two key components: primary appraisal, which assesses the degree of threat posed by the situation, and secondary appraisal, which evaluates whether the individual has sufficient resources to cope with the stressor (Lazarus and Folkman, 1984).

According to this theory, individuals perceive stressors based on their cognitive appraisals, which are influenced by their sense of control over the stressor. Individuals with higher academic self-efficacy are more likely to evaluate stressors as challenges rather than threats, leading to fewer stress responses (Kristensen et al., 2023). Conversely, those with lower self-efficacy are more prone to perceive stressors as threats, resulting in stronger stress responses (Zhang et al., 2024). Based on this framework, the relationship between academic self-efficacy and academic stress can be further understood through the cognitive appraisal process.

However, previous research has often treated academic stress as a result of academic self-efficacy, with less focus on how self-efficacy influences the occurrence and management of stress (Schunk, 1991). Some studies have found that students with high academic self-efficacy are more adaptable when facing academic stress, often viewing it as a challenge and effectively regulating their stress levels (Kristensen et al., 2023). Other research indicates a negative correlation between academic self-efficacy and academic stress, suggesting that students with higher self-efficacy experience less academic stress (Liu et al., 2024). These findings highlight the significant role of coping strategies in the relationship between academic self-efficacy and academic stress (Alkhawaldeh et al., 2023). However, the mechanisms through which academic self-efficacy affects stress via coping strategies remain underexplored.

*Coping Theory* offers additional support for this research. According to Folkman and Moskowitz, individuals typically choose different coping strategies when encountering stress, which can be either positive or negative (Folkman and Moskowitz, 2000). Positive strategies, such as seeking social support, adjusting goals, and reframing attitudes, help alleviate perceived stress, improve emotional regulation, and enhance problem-solving abilities (Teixeira et al., 2022). Negative strategies, such as avoidance or escaping from stressors, may intensify stress responses and hinder effective stress management. Individuals with high academic self-efficacy are more likely to adopt positive coping strategies, such as seeking support or adjusting their approach, to effectively address academic stress (Bandura and Wessels, 1997). In contrast, those with lower self-efficacy may resort to negative coping strategies, such as avoidance, exacerbating their stress (Endler and Parker, 1990).

Based on the theoretical foundations above, this study proposes the following hypothesis: *Coping strategies mediate the relationship between academic self-efficacy and academic stress*. Specifically, students with high academic self-efficacy are more likely to adopt positive coping strategies, thereby reducing their academic stress.

By testing this hypothesis, this research not only enhances the framework for studying the relationship between academic self-efficacy and academic stress but also offers a new theoretical and practical perspective for mitigating academic stress. Understanding how academic self-efficacy influences stress through coping strategies is crucial for designing effective educational interventions, improving students’ coping skills, and alleviating academic stress.

## Methods

### Participants

This study included a total of 3,137 middle and high school students from the Chinese regions of Inner Mongolia, Henan, and Chongqing, using a simple random sampling method between October and December 2023. In the Chinese education system, middle school students typically encompass those in Grades 7 through 9 (ages approximately 12–15), while high school students include those in Grades 10 through 12 (ages approximately 16–18). These grade levels correspond to the junior and senior stages of secondary education. The sample thus spans the full spectrum of secondary education, providing a representative understanding of the phenomena across different developmental stages and diverse geographical areas within China.

A total of 3,137 questionnaires were distributed and collected. After excluding invalid, duplicate, and logically incorrect responses, 2,720 valid questionnaires were obtained, with an effective recovery rate of 86.7%. Logical errors primarily included extreme response patterns, such as selecting the same option for all questions or providing conflicting answers to opposing items; responses outside the target range, such as unreasonable values for age, weight, or height; and inconsistencies between reverse-scored and positively scored items, which made it impossible to verify the credibility of the responses. These errors were likely due to inattentive answering, misinterpretation of the questions, or intentional errors, and their exclusion significantly improved the accuracy and reliability of the data analysis.

Among the respondents, 1,336 were boys (49.1%) and 1,384 were girls (50.9%), with ages ranging from 11 to 18 years and an average age of 14.48 ± 1.47 years. This study was approved by the Ethics Committee of *Capital University of Physical Education and Sports*, and permission was obtained from the surveyed schools. The questionnaire survey was conducted after obtaining informed consent from all participating students and their parents.

## Instruments

### Demographic characteristics

Including gender, only-child status, grade, family residence, boarding status, height, and weight.

### Academic self-efficacy scale (ASS)

The Chinese version of the Academic Self-Efficacy Scale, originally developed by Pintrich and De Groot (1990) and revised by Liang (2002), was used to assess middle school students’ academic self-efficacy. The scale consists of two dimensions: self-efficacy in learning abilities and self-efficacy in learning behaviors, with 22 items in total (11 items per dimension). A 5-point Likert scale was used, ranging from “1” (not at all true) to “5” (completely true). In this study, the Cronbach’s alpha coefficient for the scale was 0.897, and the coefficients for the two dimensions were 0.927 and 0.712, respectively.

### Academic pressure scale (APS)

The Academic Pressure Scale from the Chinese Mental Health Scale for Middle School Students was used to assess academic stress (Wang and Xinhua, 2002). The scale includes six items corresponding to items 31, 33, 36, 38, 40, and 55 of the original questionnaires. A 5-point Likert scale was used, ranging from “1” (never) to “5” (very often). Higher scores indicate greater academic stress. In this study, the Cronbach’s alpha coefficient for the scale was 0.9.

### Simplified coping style questionnaire (SCSQ)

The Simplified Coping Style Questionnaire, developed by Xie (1998), was used to assess coping styles. The scale includes two dimensions, with a total of 20 items: 12 items for positive coping and eight items for negative coping. A 4-point scale was used, ranging from “0” (never used) to “3” (frequently used). The average scores for each dimension were calculated, with higher scores indicating a tendency to use that coping style. In this study, the Cronbach’s alpha coefficient for the scale was 0.827.

### Quality control

The survey was conducted using an online platform, with a QR code and access link generated for the questionnaire. The research team conducted trial fills and verification to adjust and improve the questionnaire content. The survey was distributed with the help of school administrative staff, who were informed of the study’s purpose. After data collection, the research team conducted a second screening of the returned questionnaires, removing incomplete or invalid responses, and used double entry to record the valid data into statistical analysis software.

### Data processing and statistical analysis approach

SPSS 26.0 and Amos 26.0 statistical software were used for data management and analysis. Prior to the formal data analysis, common method bias was tested using SPSS 26.0 and Amos 26.0. Formal data analysis involved descriptive statistics and correlation analyses conducted in SPSS 26.0, with count data presented as frequencies and percentages. Amos 26.0 was then employed to test the hypothesized mediation model through latent variable structural equation modeling (SEM), with the bootstrap method set to 5,000 iterations to explore the relationships among academic self-efficacy, simplified coping styles, and academic stress. The significance level was set at *α* = 0.05.

## Results

### Common method bias testing

Since the data for this study were collected through self-reports from participants, there is a potential risk of common method bias. To minimize this bias during the administration of the survey to middle school students, the researchers emphasized the anonymity and confidentiality of the questionnaire, explicitly stated that the data would be used exclusively for scientific research purposes, and incorporated reverse-coded items into the questionnaire as a control measure.

Harman’s single-factor test was employed to assess common method bias. The analysis revealed that, in the unrotated factor solution, eight factors had eigenvalues greater than 1, with the first factor accounting for only 27.31% of the total variance, which is below the critical threshold of 40%. Additionally, a single-factor confirmatory factor analysis (CFA) was conducted using all self-reported items to further examine common method bias (Podsakoff et al., 2003). The results indicated poor model fit: *χ*^2^/df = 99.94, CFI = 0.67, GFI = 0.74, AGFI = 0.62, NFI = 0.67, and RMSEA = 0.19. Therefore, this study did not exhibit serious common method bias issues (Zhou and Long, 2004).

### Comparison of scores on various scales among students with different demographic variables

Table 1 reports the scores on various scales among students from different educational stages and family residences. From the perspective of educational stages, there were significant differences between junior high school students and senior high school students in academic self-efficacy, academic stress, positive coping style, and negative coping style (*p* < 0.01). Specifically, senior high school students scored higher on academic stress and negative coping style than junior high school students, while they scored lower on academic self-efficacy and positive coping style. From the perspective of family residence, there were no significant differences in academic self-efficacy, academic stress, and negative coping style between urban and rural students, but urban students scored significantly higher on positive coping style than rural students (*p* < 0.01).

**Table 1:** Comparison of scores on various scales among students from different educational stages and family residences ( $\bar{x}\pm s$ ) (*n* = 2,720).

Group	Number	Statistic	Academic self-efficacy	Academic stress	Positive coping style	Negative coping style
Educational stage						
Junior high	1860		77.55 ± 11.69	13.30 ± 5.33	1.84 ± 0.56	0.98 ± 0.57
Senior high	860		74.98 ± 12.15	16.08 ± 5.61	1.75 ± 0.57	1.10 ± 0.58
		*t-*value	5.27***	−12.45***	4.22***	−4.98***
Family residence						
Urban	2,164		76.96 ± 11.93	14.08 ± 6.00	1.83 ± 0.56	1.02 ± 0.58
Rural	556		75.87 ± 11.72	14.57 ± 5.44	1.73 ± 0.56	1.03 ± 0.56
		*t-*value	1.93	−1.86	3.71***	−0.44

*p < 0.05, **p < 0.01, ***p < 0.001.

### The relationship between academic self-efficacy, coping styles, and academic stress

Academic stress among middle school students was significantly negatively correlated with academic self-efficacy and positive coping style (*r* values were −0.37 and −0.3, respectively, both *p* < 0.001), and positively correlated with negative coping style (*r* = 0.32, *p* < 0.001). Positive coping style was significantly positively correlated with academic self-efficacy (*r* = 0.49, *p* < 0.001), while negative coping style was significantly negatively correlated with academic self-efficacy (*r* = −0.05, *p* < 0.01), provide a basis for subsequent hypothesis testing (Table 2).

**Table 2:** Correlation between academic stress, academic self-efficacy, positive coping style, and negative coping style (*r*).

Item	*x* ± *s*	Academic stress	Academic self-efficacy	Positive coping style	Negative coping style
Academic stress	76.74 ± 11.89	–			
Academic self-efficacy	14.18 ± 5.57	−0.37***	–		
Positive coping style	1.81 ± 0.56	−0.3***	0.49***	–	
Negative coping style	1.02 ± 0.58	0.32***	−0.05**	0.11***	–

*p < 0.05, **p < 0.01, ***p < 0.001.

### The mediating effect of coping styles between academic self-efficacy and academic stress

**Figure 1:**
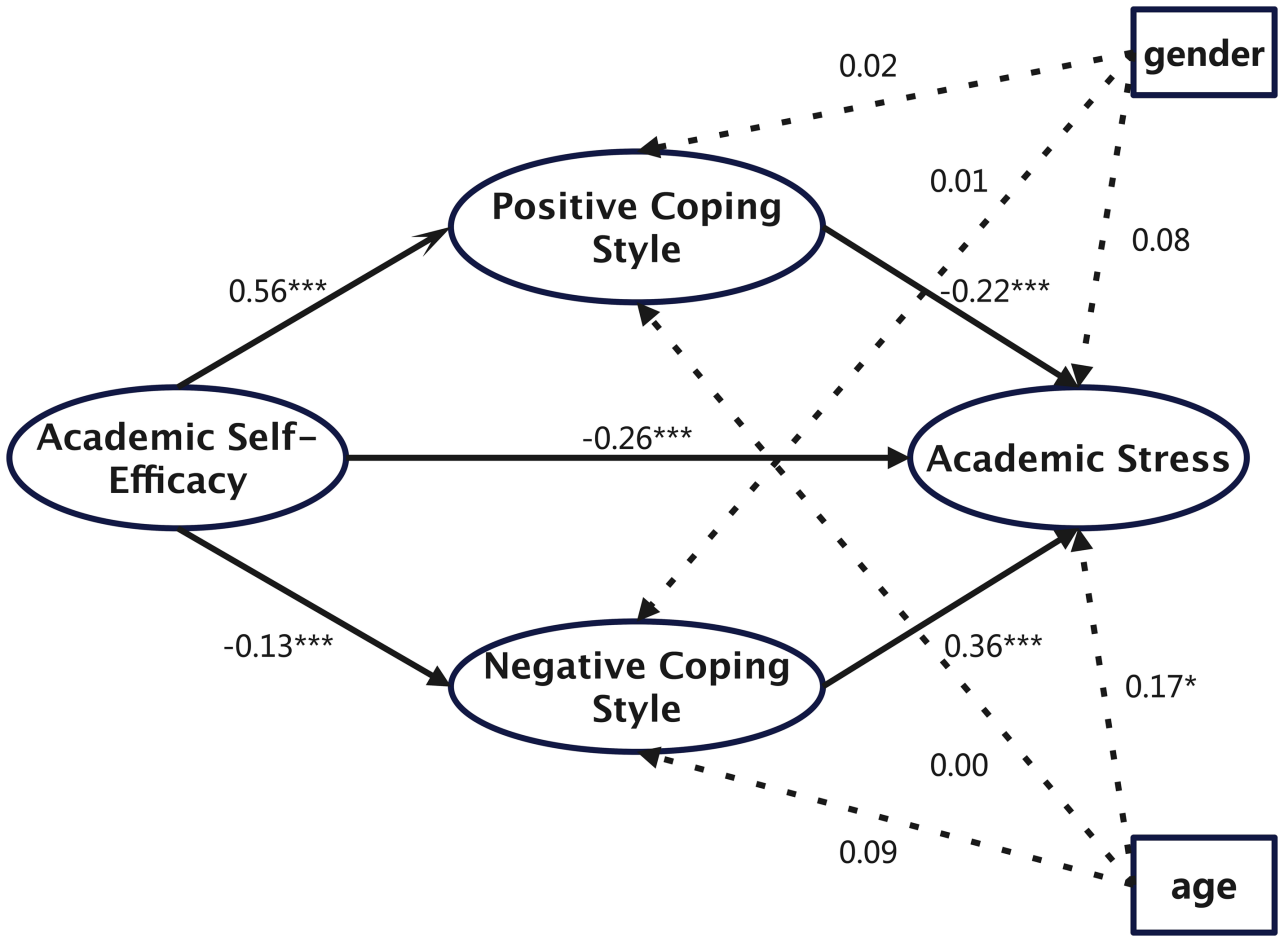
The mediating effect model of coping style between academic self-efficacy and academic stress of middle school students.

Using latent variable structural equation modeling, the relationships among variables were further examined. Demographic variables, primarily gender and age, were first controlled, and the total effect of academic self-efficacy on academic stress was found to be significant. Subsequently, mediating variables (positive coping and negative coping) were incorporated into the model. The model’s fit indices indicated an overall good fit, with all metrics showing highly satisfactory results except for *χ*^2^/df, which was slightly larger due to its sensitivity to sample size (Abd-El-Fattah, 2010; Kline, 2016; Kenny and McCoach, 2003). The specific indices were as follows: *χ*^2^/df = 21.72 (*p* < 0.001), CFI = 0.92, GFI = 0.92, AGFI = 0.88, NFI = 0.91, RMSEA = 0.08, SRMR = 0.05. Standardized path analysis results demonstrated that all paths in the model were statistically significant (see Figure 1).

The bootstrap procedure was employed to test the mediation effects, with a resampling size of 5,000 to estimate the 95% confidence intervals for the mediation effects. Results showed that academic self-efficacy significantly predicted positive coping, negative coping, and academic stress (*β* = 0.56, *p* < 0.001; *β* = −0.13, *p* < 0.001; *β* = −0.26, *p* < 0.001). Both positive and negative coping significantly predicted academic stress (*β* = −0.22, *p* < 0.001; *β* = 0.36, *p* < 0.001).

The mediation effects were generated through two pathways: academic self-efficacy → positive coping → academic stress and academic self-efficacy → negative coping → academic stress, accounting for 47.38 and 18% of the total effect, respectively. The 95% confidence intervals for both paths did not include zero, indicating statistical significance. These findings support the hypotheses of this study.

Furthermore, the standardized indirect effects were *β* = −0.12 for the positive coping pathway and *β* = 0.05 for the negative coping pathway. According to conventional benchmarks (Cohen, 2013; Preacher and Kelley, 2011), the former represents a medium effect and the latter a small effect. This suggests that while both coping styles are significant mediators, positive coping plays a relatively stronger role in linking academic self-efficacy to academic stress.

Figure 1 illustrates the structural mediation model tested in this study. As shown in the figure, academic self-efficacy not only exerts a direct effect on academic stress but also influences it through indirect pathways.

## Discussion

This study constructed a mediation model to explore the mechanism through which academic self-efficacy indirectly influences academic stress via coping styles among middle school students. The findings indicate that academic self-efficacy not only directly predicts lower academic stress but also indirectly affects it through coping styles. These results provide new theoretical insights into the relationship between academic self-efficacy and academic stress. Moreover, significant differences were observed in academic self-efficacy, coping styles, and academic stress across students of different grade levels, regions, and genders. These differences offer important implications for understanding academic stress in middle school students and devising targeted intervention strategies.

### Differences in academic stress by age and region

The study found significant differences in academic stress among students of different ages and regions. Descriptive statistics revealed that high school students experienced significantly higher academic stress compared to junior high school students (Liu, 2016). This aligns with previous findings and can be attributed to the increased complexity of academic tasks in high school (Liu and Lu, 2011), the pressure of university entrance exams, and the heightened competition for limited spots at prestigious universities. High school students face stricter evaluation standards, more demanding coursework, and an intense extracurricular training environment, all of which contribute to elevated academic stress.

In terms of regional differences, middle school students in urban areas were found to adopt more positive coping strategies than those in rural areas. This disparity may stem from the advantages of educational resources in urban regions, including high-quality teaching staff, diverse extracurricular activities, and higher parental education levels (Zhao and Yuchun, 2019). These factors provide urban students with greater psychological support and learning resources, enabling them to approach academic stress with a more positive attitude. Therefore, when addressing academic stress among middle school students, it is essential to consider urban–rural disparities, particularly the academic stress levels, coping strategies, and potential impacts on the mental health of students in rural areas.

### Academic self-efficacy as a negative predictor of academic stress

The results of this study demonstrate that academic self-efficacy significantly predicts lower academic stress. Students with high academic self-efficacy are more likely to believe in their ability to effectively handle academic tasks, displaying stronger emotional regulation skills (Honicke and Broadbent, 2016). This confidence enables them to view challenges as opportunities for growth rather than threats, thereby reducing their perceived levels of academic stress (Bandura and Wessels, 1997; Luo et al., 2023). Furthermore, students with high academic self-efficacy tend to make better use of external resources, such as teacher guidance, peer support, and family care (An et al., 2023). These sources of social support not only alleviate stress but also enhance academic achievement and intrinsic motivation (Wang and Eccles, 2013). In contrast, students with low academic self-efficacy often lack the belief in their ability to succeed, which can lead to feelings of helplessness and anxiety when faced with challenges. Such students are more likely to attribute failures to their lack of ability, reinforcing a cycle of stress and negative emotions. For example, they may give up easily when encountering difficulties, showing a lack of initiative and persistence in problem-solving (Achmad et al., 2023). This passivity leads to reduced effort and resource utilization in academics, creating a downward spiral (Frese and Fay, 2001). Negative attributions further amplify the adverse psychological impact of academic stress, affecting mental health (Usher and Pajares, 2008). Therefore, enhancing students’ academic self-efficacy serves as an effective psychological intervention, helping mitigate the detrimental effects of academic stress on mental wellbeing.

### The psychological mechanisms linking academic self-efficacy and academic stress

From a psychological perspective, academic self-efficacy, defined as an individual’s belief in their abilities within an academic context directly influences how students cognitively appraise and respond to academic challenges. According to the theory of stress appraisal (Lazarus and Folkman, 1984), an individual’s evaluation of a stressful situation determines their choice of coping strategies, with academic self-efficacy playing a pivotal role in this process (Bandura and Wessels, 1997; Pajares, 2002). Students with high academic self-efficacy tend to perceive academic challenges as manageable tasks rather than threats. This positive cognitive orientation encourages them to adopt more effective coping strategies, such as problem-solving and seeking support, thereby mitigating the negative effects of academic stress. Additionally, these students are more likely to use stress as motivation, enhancing their self-regulation abilities over time.

Conversely, students with low academic self-efficacy often lack confidence in their ability to succeed and perceive academic tasks as insurmountable challenges. They are more likely to adopt negative coping strategies, such as avoiding problems or venting emotions, which not only fail to address academic challenges but can also lead to the accumulation of stressors, creating a vicious cycle (Gao, 2023). For instance, when students avoid exams or procrastinate on completing tasks, the resulting backlog of academic work can intensify feelings of guilt and anxiety, further exacerbating their academic stress (Jagiello et al., 2024; Krispenz et al., 2019). This maladaptive coping approach acts as an amplifier of academic pressure. In contrast, enhancing academic self-efficacy can effectively break this cycle by fostering a sense of control and promoting more constructive responses to stress.

Research further demonstrates that coping strategies partially mediate the relationship between academic self-efficacy and academic stress, providing empirical support for the effectiveness of coping strategies as an intervention tool. Specifically, students with high academic self-efficacy are more likely to adopt positive coping strategies, which buffer the effects of stress by reducing its subjective perception and enhancing emotional adaptability. This finding aligns with existing research supporting the stress-buffering hypothesis of coping strategies (Compas et al., 2001; Folkman and Moskowitz, 2000; Skinner and Zimmer-Gembeck, 2007). However, it is worth noting that the mediating effect of coping strategies accounts for only part of the total effect. This suggests that, beyond coping strategies, academic self-efficacy may indirectly influence academic stress through other mechanisms. One such mechanism could be social support (Rueger et al., 2008). Students with high academic self-efficacy often exhibit stronger social skills, enabling them to actively seek assistance from teachers, parents, and peers. This social support system significantly alleviates academic stress through emotional encouragement and academic guidance (Malecki and Demaray, 2006; Wentzel, 1998).

The findings have important practical implications for alleviating academic stress among secondary school students. First, schools should focus on enhancing students’ academic self-efficacy by setting individualized academic goals, providing constructive feedback, and organizing interdisciplinary learning projects. These efforts can strengthen students’ sense of control and achievement in their academic tasks. Particularly in high school, teachers should assist students in creating effective time management plans and guide them to break down academic goals into achievable steps, thereby reducing the psychological burden of large-scale objectives. Second, the mediating role of coping strategies underscores the importance of mental health education in schools. Programs should incorporate scenario-based training and psychological counseling to cultivate positive coping strategies such as problem-solving, emotion regulation, and seeking social support while minimizing maladaptive behaviors. Finally, families and communities should serve as critical support systems for relieving academic stress. Parents need to provide both academic guidance and emotional support, encouraging children to share their difficulties and offering reassurance. At the societal level, optimizing resource allocation can help provide students in rural areas with greater access to quality educational resources and mental health services, thereby strengthening their ability to manage academic stress. Through the collaborative efforts of schools, families, and communities, the adverse effects of academic stress on students’ mental health can be effectively mitigated, fostering an environment conducive to the holistic development of secondary school students.

### Implications of the study

This study explores the relationships among academic self-efficacy, coping strategies, and academic stress, emphasizing how academic self-efficacy shapes students’ approaches to managing stress. The findings demonstrate that students with high academic self-efficacy are more likely to view stress as a challenge and adopt positive coping strategies, such as problem-solving, which alleviate stress and enhance resilience. Conversely, low self-efficacy leads to negative coping, exacerbating stress.

Coping strategies were also found to mediate the relationship between academic self-efficacy and academic stress, indirectly reducing stress and improving emotional regulation and academic outcomes. Moreover, the role of social support as a potential pathway warrants further exploration, as students with higher self-efficacy are more inclined to seek help from teachers, parents, and peers.

In conclusion, fostering academic self-efficacy and promoting effective coping strategies are critical for reducing stress and improving academic performance, offering valuable insights for educational interventions.

### Limitations of the study

Although this study provides valuable insights into the relationships among academic self-efficacy, coping strategies, and academic stress, several limitations should be addressed. First, the cross-sectional design reveals correlations but does not establish causal relationships. Future research should adopt longitudinal or experimental designs to explore how enhancing academic self-efficacy causally impacts academic stress.

Second, the findings on coping strategies’ mediating role are based on statistical analyses of survey data, which may be influenced by confounding variables and individual differences. Experimental studies, such as randomized controlled trials, are needed to confirm these effects and assess the impact of interventions targeting positive coping strategies.

Third, this study did not include other potentially important variables, such as family environment, parental expectations, or social support, which may also influence students’ academic stress. In the Chinese cultural context, Confucian academic pressure and filial piety may further shape how students perceive and respond to academic demands, while urban–rural disparities in educational resources and social support may lead to differences in self-efficacy, coping strategies, and stress experiences. Future research should therefore consider incorporating these contextual and cultural factors into the models to provide a more comprehensive understanding of the mechanisms involved.

Finally, the sample, primarily drawn from specific regions, limits the generalizability of the results. Differences in educational resources, cultural contexts, and social support across regions may affect academic stress. Future research should broaden the sample scope, employ multi-level analyses, and conduct longitudinal studies to uncover more generalizable patterns and effective interventions.

## Author’s note

In China’s education system, where academic success is paramount, the surge in academic stress among middle school students has become a significant concern. This study investigates the pivotal role of coping styles in mediating the relationship between students’ academic self-efficacy and the stress they encounter. Given the high stakes of academic performance and its implications for students’ futures, understanding how they cope with stress is essential. The research is particularly salient in the current educational climate, where reforms aim to reduce students’ burdens and emphasize a well-rounded educational experience. Identifying the interplay between self-efficacy, coping strategies, and stress can inform the development of supportive interventions and educational strategies that prioritize student mental health alongside academic achievement. This work is critical for educators and policymakers striving to craft a learning environment conducive to student resilience. By examining the mechanisms through which students handle academic pressure, we can better support their ability to navigate the challenges of the educational system, ultimately enhancing their capacity to succeed academically while maintaining their wellbeing.

## Data Availability

The raw data supporting the conclusions of this article will be made available by the authors, without undue reservation.
